# The Impact of Type 2 Diabetes on Peripheral and Cerebral Hemodynamic Responses to Active Stand

**DOI:** 10.1093/gerona/glae073

**Published:** 2024-03-04

**Authors:** Belinda Hernández, Adam H Dyer, Ciaran Finucane, Bernardo Nipoti, Roman Romero-Ortuno, Richard Reilly, Rose Anne Kenny

**Affiliations:** The Irish Longitudinal Study on Ageing, Department of Medical Gerontology, School of Medicine, Trinity College Dublin, Dublin, Ireland; Discipline of Medical Gerontology, School of Medicine, Trinity College, Dublin, Ireland; Discipline of Medical Gerontology, School of Medicine, Trinity College, Dublin, Ireland; Department of Age-Related Healthcare, Tallaght University Hospital, Dublin, Ireland; Discipline of Medical Gerontology, School of Medicine, Trinity College, Dublin, Ireland; Mercer’s Institute for Successful Ageing, St. James’s Hospital, Trinity College, The University of Dublin, Dublin, Ireland; Department of Economics, Management, and Statistics, University of Milano-Bicocca, Milan, Italy; Discipline of Medical Gerontology, School of Medicine, Trinity College, Dublin, Ireland; Mercer’s Institute for Successful Ageing, St. James’s Hospital, Trinity College, The University of Dublin, Dublin, Ireland; School of Engineering, Trinity College, The University of Dublin, Dublin, Ireland; Trinity Centre for Biomedical Engineering, Trinity College, The University of Dublin, Dublin, Ireland; The Irish Longitudinal Study on Ageing, Department of Medical Gerontology, School of Medicine, Trinity College Dublin, Dublin, Ireland; Discipline of Medical Gerontology, School of Medicine, Trinity College, Dublin, Ireland; (Medical Sciences Section)

**Keywords:** Active stand, Cerebral perfusion, Functional regression, Orthostasis, Type 2 diabetes

## Abstract

**Background:**

Although type 2 diabetes mellitus (T2DM) is an established risk factor for cognitive impairment, the underlying mechanisms remain poorly explored. One potential mechanism may be through effects of T2DM on cerebral perfusion. The current study hypothesized that T2DM is associated with altered peripheral and central hemodynamic responses to orthostasis, which may in turn be associated with cognitive impairment in T2DM.

**Methods:**

A novel use of function-on-scalar regression, which allows the entire hemodynamic response curve to be modeled, was employed to assess the association between T2DM and hemodynamic responses to orthostasis. Logistic regression was used to assess the relationship between tissue saturation index (TSI), T2DM, and cognitive impairment. All analyses used cross-sectional data from Wave 3 of The Irish Longitudinal Study on Ageing (TILDA).

**Results:**

Of 2 984 older adults (aged 64.3 ± 8.0; 55% female), 189 (6.3%) had T2DM. T2DM was associated with many features that are indicative of autonomic dysfunction including a blunted peak heart rate and lower diastolic blood pressure. T2DM was associated with reduced TSI and also with greater odds of impaired performance on the Montreal Cognitive Assessment (odds ratio [OR]: 1.62; confidence interval [CI: 1.07, 2.56]; *p* = .019). Greater TSI was associated with lower odds of impaired performance (OR: 0.90, CI [0.81–0.99]; *p* = .047).

**Conclusions:**

T2DM was associated with impaired peripheral and cerebral hemodynamic responses to active stand. Both T2DM and reduced cerebral perfusion were associated with impaired cognitive performance. Altered cerebral perfusion may represent an important mechanism linking T2DM and adverse brain health outcomes in older adults.

Type 2 diabetes mellitus (T2DM) affects 1 in 10 older adults, the prevalence of which is increasing (1). Although T2DM is associated with a greater risk of cognitive impairment, dementia, and adverse brain health outcomes in later life (2–4), the underlying mechanisms remain poorly understood. An important putative mechanism linking diabetes and adverse brain health outcomes in older adults is impaired cerebral perfusion, which has been previously linked to both cognitive impairment and dementia (5,6).

T2DM is associated with an increased risk of orthostatic hypotension (OH) (7), the risk of which increases with the severity and duration of T2DM (8–12). A meta-analysis has estimated a prevalence of 24% of those with T2DM have OH, although estimates vary widely (13). In most of these studies, OH is defined using sphygmomanometer-based measurements, assessing blood pressure during either seated-to-stand or lying-to-stand maneuver with measurements made pre-stand and 1-minute intervals for 3 minutes post-stand. Noninvasive continuous beat-to-beat blood pressure using a Finometer during active stand offers a more sensitive assessment of OH phenotypes, and may capture more subtle impairments in orthostatic blood pressure behavior resulting from autonomic dysfunction in T2DM (8,14).

Importantly, OH is associated with numerous adverse health outcomes in older adults including future falls/syncope (15), stroke (16), depression (17), cardiovascular disease (13,18), cognitive decline (19), and even mortality (20). Because T2DM is independently associated with many of these, impaired orthostatic blood pressure responses/OH and autonomic dysfunction may represent an important mechanism linking T2DM and these outcomes in older adults. Although 1 small study has demonstrated poorer cognitive performance in individuals with diabetes and OH versus those with diabetes but without OH (21), the relationship between subtle impairments in peripheral hemodynamic responses and cognitive impairment has not been explored in T2DM.

In OH, it is hypothesized that failure/impaired recovery of blood pressure on standing may lead to impaired cerebral oxygenation. Several studies have examined the relationship between diabetes and cerebral blood flow (22,23), typically measured using arterial spin labeling, magnetic resonance imaging (23–25), or single-photon emission computed tomography (26). Evidence from these studies highlights cerebral perfusion alterations in the occipital lobe, areas involved in the default mode network, and the cerebellum in T2DM (27). Some small studies in older adults with diabetes have linked cerebral perfusion abnormalities to poorer cognitive performance (28–30) and gait speed decline (31,32). However, no studies have examined the impact of T2DM on the dynamics of cerebral oxygenation during active stand in older adults with T2DM or the potential relationship between peripheral hemodynamic responses (orthostatic blood pressure behavior/OH), cerebral oxygenation, and cognitive impairment in T2DM.

Although previous approaches to estimate cerebral oxygenation have required neuroimaging and tracers, near-infrared spectroscopy (NIRS) has emerged as an important noninvasive surrogate marker of cerebral oxygenation (33–35). Using a probe attached to the forehead, NIRS systems measure the ratio between oxygenated and total hemoglobin, giving an estimate of tissue saturation index (TSI) (36,37). Such objective measurements of cerebral oxygenation, reflecting cerebral perfusion, have been shown to be associated with syncope, depression, and multimorbidity in older adults (38–41). However, the impact of T2DM on cerebral oxygenation measured using NIRS has not been explored to date.

Here, we examined the impact of T2DM on (i) peripheral hemodynamic responses (noninvasive continuous beat-to-beat measurement using a Finometer) and (ii) cerebral oxygenation responses (using NIRS) to active standing in older adults. We further examined whether subtle impairments in transient peripheral and cerebral hemodynamic responses were associated with cognitive impairment in older adults, and the modulatory impact of T2DM on this association.

## Method

### Study Approval, Setting, and Participants

Data were obtained cross-sectionally from Wave 3 (2014–2015) of The Irish Longitudinal Study on Ageing (TILDA), a prospective nationally representative study of community-dwelling older adults in Ireland. Participants with a doctor’s diagnosis of dementia and/or cognitive impairment were excluded at Wave 1 as they could not provide consent to participate in the study. All data from the active stand test were obtained in a health center with assessments administered by trained nurses (42,43).

The study was approved by the Faculty of Health Sciences Research Ethics Committee at Trinity College Dublin and adhered to the Declaration of Helsinki.

### Measurement of Peripheral and Central Hemodynamic Responses to Orthostasis

The active stand protocol is described in Supplementary Appendix 1. Briefly, continuous beat-to-beat measurements of systolic/diastolic blood pressure (SBP/DBP), heart rate (HR), total peripheral resistance (TPR), stroke volume (SV), and cardiac output (CO) were measured using a Finometer MIDI device, and TSI was measured using a NIRS device. The validity and reliability of these devices are discussed in (44,45).

All hemodynamic signals were analyzed as changes from supine baseline (where supine baseline is the mean value 60–30 seconds before standing). Therefore, positive values indicate an increase from supine baseline and negative a decrease from supine baseline. Additionally, absolute HR and TSI were reported as, unlike the other hemodynamic responses, these signals can be reliably measured in absolute terms.

SV and CO were normalized by body surface area prior to analysis. Therefore, these signals describe the change from supine baseline per square meter of body surface area and will be referred to as the stroke volume index (SVI) and cardiac index (CI) henceforth.

### Participant Characteristics and Exclusion Criteria

Overall, 2 984 participants were included in the analyses of peripheral hemodynamic signals (using the Finometer) and 2 496 for central hemodynamic signals (using NIRS). See Supplementary Appendix 2 and Supplementary Table 1 for a full list of exclusion criteria.

## Variable Definitions

### Diabetes Status

T2DM was identified using the following objective criteria: (i) HbA_1c_ levels ≥48 mmol/mol as per the American Diabetes Association (ADA) criteria (1) and/or (ii) use of any of the following antidiabetic medications as per the WHO Anatomical Therapeutic Classification system (ATC) codes: insulins and analogs (A10A), blood-glucose-lowering drugs excluding insulins (A10B), or other diabetes drugs (A10X). Participants using insulins and analogs (A10A) in isolation were excluded (*n* = 1) as they were suspected to have type 1 diabetes (insulin-dependent diabetes mellitus). Further, pre-diabetes was defined as HbA_1c_ levels ≥39 mmol/mol and <48 mmol/mol (and not taking any diabetes medication) as per ADA criteria and as described in (2). Self-reported doctor’s diagnosis was not included in these criteria as the survey item in question asked participants whether they had ever been diagnosed with “diabetes or high blood sugar” and so the wording was considered too ambiguous to distinguish between pre-diabetes and diabetes.

### Confounders

The following variables were controlled in multivariate analyses: age, sex, education, smoking history, physical activity, cardiovascular diseases, comorbidities, disabilities, waist–height ratio, pulse wave velocity, use of antidepressants/antipsychotic and/or anxiolytic medication, use of lipid-modifying drugs, use of beta-blockers, use of calcium channel blockers, and use of other antihypertensives. Full definitions of all outcomes and confounding variables are given in Supplementary Appendix 3.

### Cognitive Impairment

The Montreal Cognitive Assessment (MoCA) was used as an assessment of global cognitive performance, with a score of 23 or less classified as impaired cognitive performance based on published normative population data (46).

## Statistical Analysis

### Modeling Hemodynamic Responses to Standing

**Figure 1.**
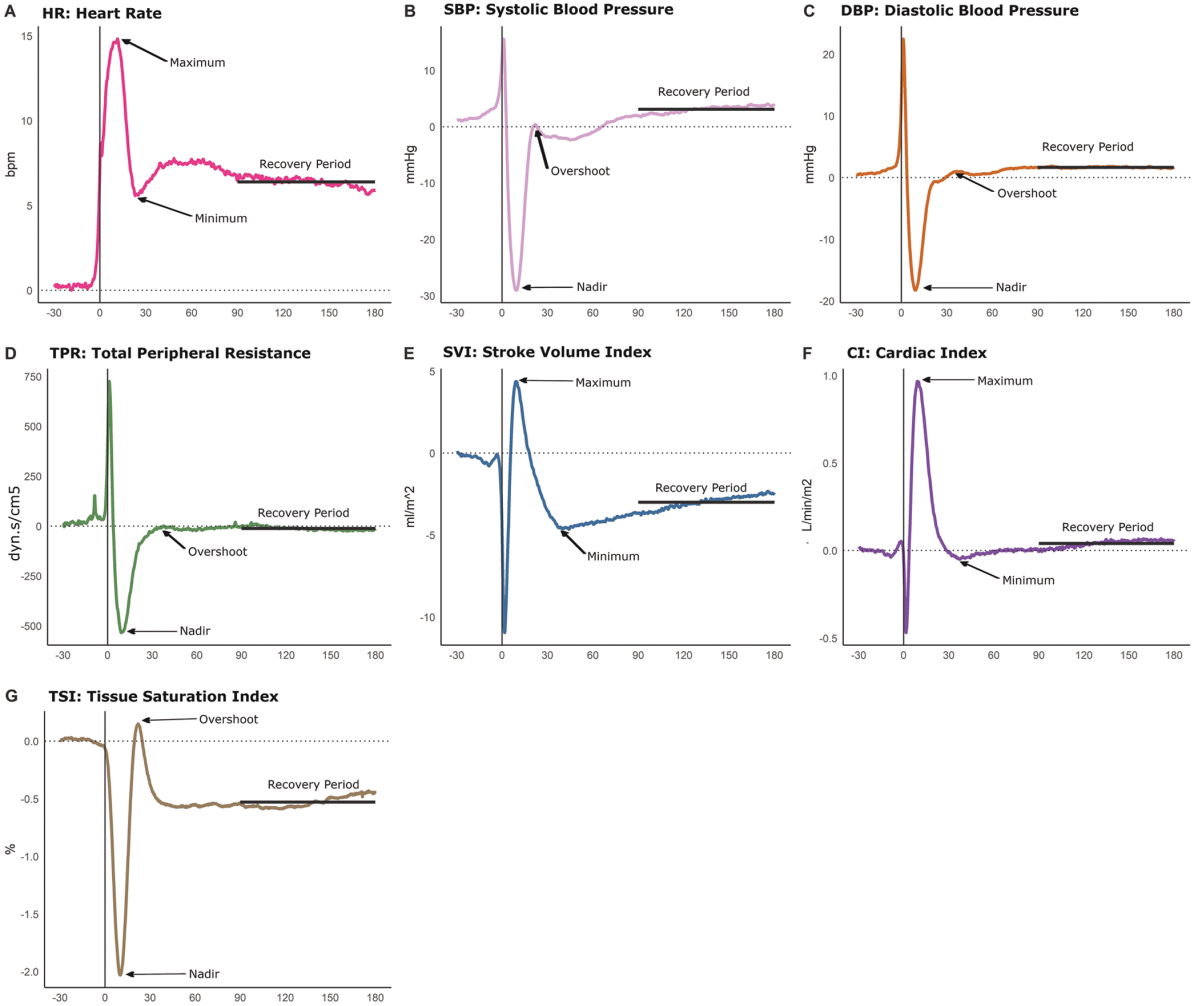
Visualization of mean peripheral and central hemodynamic responses and univariate descriptive features (*n* = 2 984, aged 64.3 ± 8.0 years). HR = heart rate; SBP = systolic blood pressure; DBP = diastolic blood pressure; TPR = total peripheral resistance; SVI = stroke volume index; CI = cardiac index; TSI = Tissue Saturation Index. All signals denote a change from supine baseline (mean from −60 to −30 seconds before stand). Therefore, each time point denotes the change from supine baseline. For SBP, DBP, and TSI, the nadir was detected as the minimum value between 5 and 40 seconds post-stand and the overshoot as the first peak after the nadir and before 60 seconds after stand. For the remaining signals, “maximum” refers to the first peak between 5 and 40 seconds after stand and “minimum” to the first nadir after maximum and before 60 seconds post-stand. Black lines denoting “Recovery period” refer to the average change from baseline between 90 and 180 seconds post-stand.

Figure 1 describes the signal features extracted for univariate analysis. These features were also described in (41). Briefly, for SBP, DBP, and TSI, the nadir was detected as the minimum value between 5 and 40 seconds post-stand and the overshoot as the first peak after the nadir and before 60 seconds after stand. For the remaining signals, “maximum” refers to the first peak between 5 and 40 seconds after stand and “minimum” to the first nadir after maximum and before 60 seconds post-stand. In all cases, the “Recovery Period” refers to the average change from baseline between 90 and 180 seconds post-stand.

In multivariable models, the response to standing of HR, SBP, DBP, TPR, SVI, CI, and TSI was modeled independently using function-on-scalar regressions with the hemodynamic curves as the response variable (47). The major advantage of using functional regression in this instance is that it allows for the independent effect of diabetes to be estimated dynamically over all time points of the active stand experiment after accounting for other confounders rather than at isolated times/features as earlier. For more details on the methodology and choice of tuning parameters, see Supplementary Appendix 4 and Supplementary Figures 1 and 2. Analysis was performed using the *fda*, *fda.usc*, and *refund* packages in R4.0.5.

All multivariable models were adjusted for age, sex, education, smoking status, pulse wave velocity, waist-to-height ratio, physical activity, presence of disabilities, history of cardiovascular diseases, medical comorbidity, and medication usage. Seated SBP was also controlled for except where SBP/DBP was the response variable.

### Assessing the Relationship Between T2DM, Hemodynamic Responses, and Cognitive Performance

Logistic regression was used to assess the relationship between cognitive impairment (outcome variable) and (i) T2DM status and (ii) mean TSI during the recovery period (90–180 seconds post-stand) as independent variables. With regards to the recovery period, Van Wijnen suggests that early stabilization of blood pressure due to standing should occur within 30 seconds post-stand (48) in healthy older adults, and therefore, it is reasonable to expect that early recovery of peripheral and central hemodynamic signals should have occurred by 90–180 seconds post-stand. Figure 1 also shows that, on average, all signals have recovered to a stable value beyond 90 seconds. In sensitivity analyses, it was found that the results were not sensitive to the time period chosen for the recovery period and conclusions remained unchanged whether using 30–180, 60–180, or 90–180 seconds.

The model assessing the relationship between T2DM and TSI recovery on cognitive impairment was adjusted for age, sex, education, comorbidities, use of antidepressants/antipsychotic and/or anxiolytic medication, seated hypertension and/or use of antihypertensive including beta-blockers and calcium channel blockers, physical activity, smoking status, presence of disabilities, and waist-to-height ratio. A second model that included the covariates mentioned earlier also further adjusted for the recovery period of the remaining peripheral hemodynamic responses (HR, SBP, DBP, SVI, CI, and TPR).

## Results

Table 1 and Supplementary Table 2 show the characteristics of the sample according to diabetes status. Those with T2DM were older, more likely to be male, less likely to have never smoked, had higher pulse wave velocity, higher waist-to-height ratio, lower levels of physical activity, higher use of antihypertensives and lipid-modifying drugs, and a higher proportion of disabilities and cardiovascular disease than those without T2DM.

**Table 1.** Characteristics of the Study Sample According to Diabetes Status

	No Diabetes, *N* = 2 435	Pre-Diabetes, *N* = 360	Diabetes, *N* = 189	*p* Value
HbA1c mean (*SD*) mmol/mol				
Age	63.8 (7.8)	66.4 (8.6)	66.9 (7.9)	<.001
Sex, *n* (%)				
* *Female	1 389 (57.1)	182 (50.7)	62 (32.8)	<.001
Education, *n* (%)				.002
Primary	384 (15.8)	72 (20.1)	42 (22.2)	
Secondary	961 (39.5)	162 (45.1)	69 (36.5)	
Third	1 087 (44.7)	125 (34.8)	78 (41.3)	
Smoking status, *n* (%)				.005
Never	1189 (48.9)	160 (44.6)	71 (37.6)	
Former	1020 (41.9)	153 (42.6)	100 (52.9)	
Current	223 (9.2)	46 (12.8)	18 (9.5)	
Pulse wave velocity (m/s)	10.2 (2.0)	10.8 (2.1)	11.3 (2.1)	<.001
Waist height ratio (healthy 0.4–0.49; low risk 0.5–0.59; high risk ≥0.6)	0.57 (0.07)	0.62 (0.08)	0.63 (0.07)	<.001
Baseline seated, SBP	132.3 (18.4)	134.2 (17.8)	135.5 (18.5)	.015
Baseline seated, DBP	81.0 (10.2)	81.0 (10.5)	79.1 (10.1)	.043
Baseline HR	64.8 (9.5)	67.4 (10.6)	70.5 (11.2)	<.001

*Notes*: See Methods section for exact definitions of the remaining variables. DBP = diastolic blood pressure; HR = heart rate; SBP = systolic blood pressure; *SD* = standard deviation.

### Peripheral and Central Responses to Active Standing in T2DM

Table 2 shows a descriptive summary of the peripheral and central hemodynamic responses according to diabetes status using the signal features depicted in Figure 1. All summaries are shown as a change from supine baseline (where supine baseline is the mean value 60–30 seconds before standing).

**Table 2.** Descriptive Univariate Summary Change From Supine Baseline to Stand in Peripheral and Central Hemodynamic Responses

	No Diabetes (*n* = 2435)	Pre-Diabetes (*n* = 360)	Diabetes (*n* = 189)	*p* Value
HR (bpm)				
Maximum	16.13 (6.47)	15.66 (6.82)	13.26 (6.05)	<.001
Minimum	5.21 (6.30)	6.05 (6.75)	5.72 (5.99)	.045
Recovery period	6.97 (6.13)	6.76 (6.30)	5.83 (5.86)	.046
SBP (mmHg)				
Nadir	−27.38 (15.48)	−26.88 (17.71)	−24.64 (15.68)	.068
Overshoot	3.19 (15.03)	2.76 (17.10)	1.83 (17.75)	.475
Recovery period	2.46 (13.79)	3.83 (13.69)	2.73 (13.19)	.487
DBP (mmHg)				
Nadir	−16.35 (9.07)	−16.15 (9.92)	−16.30 (8.87)	.928
Overshoot	3.07 (7.40)	1.90 (8.37)	−0.32 (8.46)	<.001
Recovery period	1.97 (6.42)	1.74 (6.46)	−0.40 (6.80)	<.001
SVI (mL/m^2^)				
Maximum	4.38 (7.99)	5.05 (8.41)	6.50 (8.51)	.001
Minimum	−5.42 (7.49)	−4.43 (7.56)	−2.27 (8.68)	<.001
Recovery period	−4.02 (7.39)	−2.74 (7.30)	0.39 (8.72)	<.001
CI (L/min/m^2^)				
Maximum	0.91 (0.62)	0.94 (0.62)	1.00 (0.69)	.142
Minimum	−0.13 (0.47)	−0.01 (0.48)	0.10 (0.63)	<.001
Recovery period	−0.01 (0.46)	0.07 (0.44)	0.26 (0.59)	<.001
TPR (mm Hg·min/L)				
Nadir	−538.1 (395.5)	−481.0 (305.2)	−395.40 (234.7)	<.001
Overshoot	60.5 (403.7)	−11.3 (264.5)	−47.58 (254.6)	<.001
Recovery period	10.2 (330.6)	−15.3 (269.9)	−79.1 (199.6)	<.001
TSI %				
Nadir	−1.96 (1.71)	−2.08 (1.75)	−2.50 (1.88)	<.001
Overshoot	0.15 (1.72)	0.11 (1.65)	−0.21 (1.65)	.048
Recovery period	−0.57 (2.07)	−0.47 (1.73)	−0.82 (1.82)	.228

*Notes*: All signals denote a change from supine baseline (taken as the mean from −60 to −30 seconds before stand) for heart rate (HR), systolic blood pressure (SBP), Diastolic Blood Pressure (DBP), stroke volume index (SVI), cardiac index (CI), total peripheral resistance (TPR), and Tissue Saturation Index (TSI). For SBP, DBP, and TSI, the nadir was detected as the minimum value between 5 and 40 seconds post-stand and the overshoot as the first peak after the nadir and before 60 seconds after stand. For the remaining signals, “maximum” refers to the first peak between 5 and 40 seconds after stand and “minimum” to the first nadir after maximum and before 60 seconds post-stand. Recovery period in all cases refers to the average change from baseline between 90 and 180 seconds post-stand.

In univariate analysis, T2DM was significantly associated with a reduced peak/maximum HR and lower steady-state HR during recovery. T2DM was also associated with an impaired DBP recovery at overshoot and during the steady-state recovery period. Unlike T2DM, the No Diabetes and Pre-Diabetes groups recovered to a higher value than their supine baseline. T2DM was associated with higher SVI for all signal features (denoting a larger increase from supine baseline at the maximum and recovery period and an attenuated drop from supine baseline at the minimum) and higher CI (larger increase from supine baseline) at the minimum and recovery period. With respect to TPR, T2DM was associated with a smaller drop to nadir but also impaired/lower overshoot and recovery when compared to the No Diabetes and Pre-Diabetes groups. Regarding change from supine baseline TSI, T2DM was associated with significantly lower nadir and lower overshoot in univariate analysis.

### Multivariate Analysis

**Figure 2.**
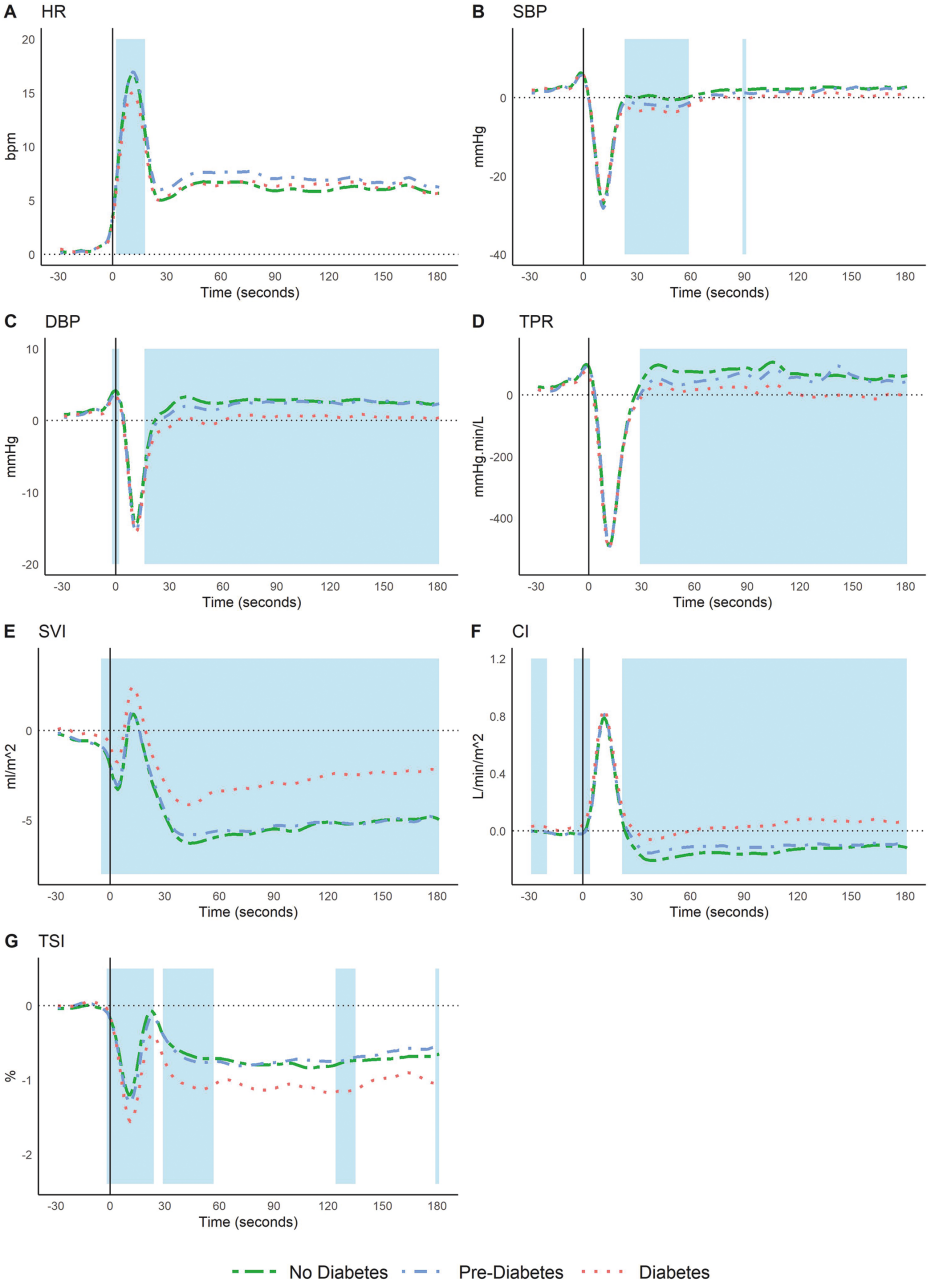
Predicted signals for No Diabetes, Pre-Diabetes, and Diabetes. Predicted signals for No Diabetes, Pre-Diabetes and Diabetes for the average TILDA participant indicating change from supine baseline in the following: (A) heart rate; (B) systolic blood pressure; (C) diastolic blood pressure; (D) total peripheral resistance; (E) stroke volume index; (F) cardiac index; (G) tissue saturation index. X-axis denotes time from 30 seconds before standing to 180 seconds after stand. Shaded regions indicate time points where the coefficient for diabetes was significant. Vertical line at Time 0 indicates the time of transition from supine position to standing. DBP, SBP, and TSI lower in T2DM. HR has blunted peak for those with T2DM. SVI and CI higher in T2DM.

Supplementary Figures 3–11 show the covariates and 95% pointwise confidence intervals (CIs) for the multivariable model of all hemodynamic signals. Figure 2 shows the predicted response curves for the average participant with respect to diabetes status for the peripheral hemodynamic measures (A–F) and TSI (G). Shaded areas indicate the time periods where the coefficient for diabetes was significant after robust covariate adjustment (ie, the time periods where the 95% CI for diabetes in Supplementary Figures 3–11 and Supplementary Appendix 5 do not cross zero).

Under multivariable models (after adjustment for important clinical confounders), T2DM was significantly associated with a blunted increase in HR between 2 and 18 seconds post-standing, corresponding to the window capturing the initial HR peak (see Figure 2A).

T2DM was associated with larger drops from supine baseline SBP from 23 to 59 seconds post-stand (Figure 2B) and a larger drop from supine baseline in DBP from 15 seconds onward (Figure 2C). T2DM was associated with significantly impaired/lower TPR from around the overshoot at 29 seconds post-stand until the end of the test (Figure 2D). T2DM was also associated with a smaller drop in SVI from 25 to 180 seconds post-stand (Figure 2E). Regarding CI, T2DM was significantly higher than no T2DM from 21 to 180 seconds post-stand. In fact, for T2DM, CI in this period was higher than at supine baseline on average whereas CI for No Diabetes and Pre-Diabetes remained lower than at supine baseline (Figure 2F).

It was found that T2DM was associated with significantly higher absolute HR throughout the active stand experiment (mean HR for No Diabetes 71.28 bpm compared to 76.11 bpm for T2DM; See Supplementary Figures 10 and 12). Given that CI is by definition SVI × HR, it can be seen that both significantly elevated absolute HR and higher SVI from supine baseline are contributing to the elevated CI in T2DM.

Regarding cerebral oxygenation, T2DM was associated with significantly lower change from supine baseline TSI from the nadir to the overshoot and TSI from 8 to 57 seconds post-stand (see Figure 2G). T2DM was associated with significantly lower absolute TSI levels throughout the test (mean TSI for No Diabetes 70.94% compared to 72.24% for T2DM; see Supplementary Figure 12).

### Hemodynamic Responses, T2DM, and Cognitive Performance

**Figure 3.**
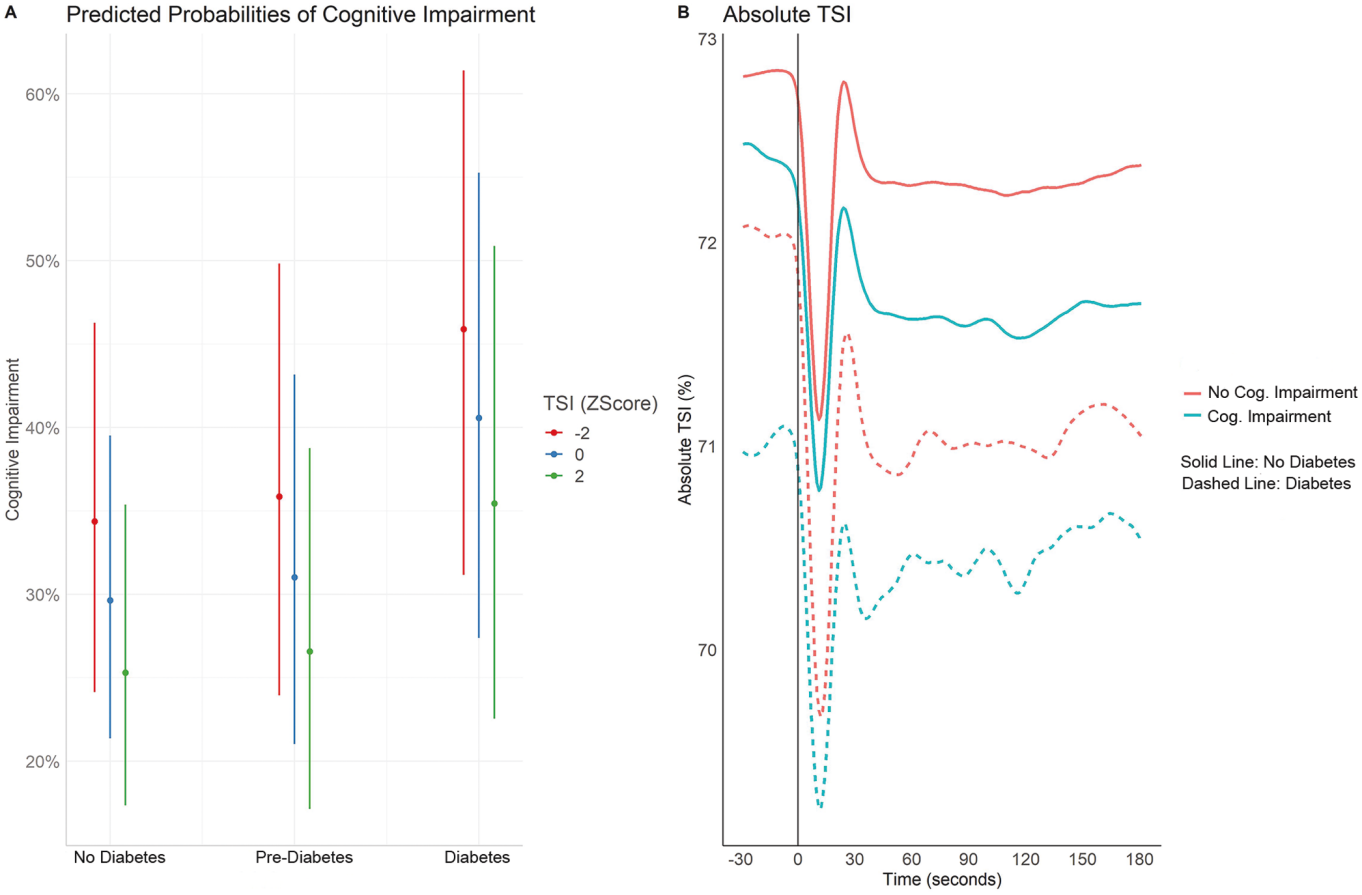
(A) Predicted probability of cognitive impairment by diabetes status and mean TSI recovery (−2 = 2 standard deviations below the mean; 0 = mean TSI; 2 = 2 standard deviations above the mean). Mean TSI recovery refers to the mean value of TSI during the recovery period 90–180 seconds post-stand. Predicted probabilities have been adjusted for age, sex, education, comorbidities, use of antidepressants/antipsychotic and/or anxiolytic medication, seated hypertension, and/or use of antihypertensive medication including calcium channel, beta-blockers, physical activity, smoking status, presence of disabilities, and waist-to-height ratio. (B) Mean TSI signal by cognitive impairment and diabetes status from highest to lowest; top solid red line (no cognitive impairment, no diabetes), second solid blue line (cognitive impairment, no diabetes), dashed red (no cognitive impairment, diabetes), bottom dashed blue line (cognitive impairment, diabetes).

Figure 3A shows the marginal predicted probabilities of cognitive impairment by diabetes status for varying levels of TSI. Figure 2B shows a visualization of mean absolute TSI throughout the active stand test by cognitive impairment and diabetes status. The corresponding odds ratios (ORs), CIs, and *p* values from the model in Figure 2A can be found in Supplementary Table 2. As can be seen, T2DM was associated with higher odds of cognitive impairment (OR 1.62; CI (1.07, 2.41); *p* = .019) as was lower TSI (OR 0.90; CI (0.81, 0.99); *p* = .047; see also Figure 3A). This was after adjustment for other confounders such as age, sex, pre-existing hypertension, use of medications, and blood pressure recovery after standing among a number of other covariates. The coefficient of T2DM was slightly attenuated from 1.67 (model not including TSI) to 1.62 after controlling for TSI (Supplementary Table 2A). As can be seen from Figure 3A, the predicted probability of cognitive impairment for the No Diabetes group was 30% CI (21%, 40%) compared to 41% CI (27%, 55%) for the Diabetes group when TSI was at its mean value. When TSI was 2 standard deviations (*SD*) below the mean, these probabilities of cognitive impairment increased to 34% CI (24%, 46%) and 46% CI (31%, 61%) for the No Diabetes and T2DM groups, respectively. There was no evidence of an interaction effect between diabetes status and TSI recovery. Figure 3B further emphasizes the fact that there is lower mean TSI in general in T2DM and that the lowest mean TSI was observed in those with cognitive impairment and T2DM. Figure 3B further shows that this association for absolute TSI was present at supine baseline and not just on standing.

The coefficient for TSI recovery and diabetes status on cognitive impairment remained unchanged and significant even after the addition of other peripheral hemodynamic responses (see Supplementary Table 2). Other signal features identified in Figure 1 were not significantly associated with cognitive impairment.

## Discussion

In the current study of almost 3 000 community-dwelling older adults, T2DM was associated with impaired HR, BP, and TPR recovery and with a reduced drop in SVI and increased CI from supine baseline to standing. T2DM was associated with impaired recovery of change from supine baseline TSI during the first minute after standing and was associated with higher absolute HR and lower absolute TSI throughout the active stand test even at supine baseline. Notably, both T2DM and lower TSI were associated with a greater likelihood of impaired cognitive function after robust covariate adjustment. These results suggest that impaired peripheral and cerebral responses to active stand may in part explain the relationships between T2DM and cognitive impairment in older adults. Both T2DM and TSI recovery remained significantly associated with cognitive impairment even after adjustment for other peripheral hemodynamic measurements.

The transient orthostatic response to standing in healthy older adults has been well studied and is an important physiological stressor which requires a flexible response in peripheral and central hemodynamics to stabilize BP and protect the brain from large sudden drops in BP. Briefly, when moving from a supine to upright position, blood pools in the legs leading to a reduction in blood pressure and venous return. This drop in blood pressure is counteracted by the arterial baroreflex, leading to an initial increase in HR, SV, and CO to counteract the sudden drop in blood pressure (49,50). Similarly, cerebral oxygenation measured by TSI decreases, reaching a nadir around 10 seconds after standing before recovering (36,37). Impaired orthostatic responses to standing have previously been shown to be associated with cognitive impairment (19), depression (17), and dementia (51) and have also been shown to be present in diabetes (7). However, the independent dynamic association of T2DM and peripheral and central hemodynamic responses, to our knowledge, has not been investigated, nor has the association between subtle impairments in transient peripheral and cerebral hemodynamic responses, T2DM, and cognitive impairment.

Overall, our results demonstrate evidence of a significantly blunted HR response and significantly higher absolute HR throughout T2DM, both features known to be associated with autonomic dysfunction and poorer health outcomes (52). T2DM was also associated with a larger drop in DBP and TPR after standing. These results suggest that autonomic impairment, as a result of T2DM may be leading to impairment of the TPR response to standing in diabetes and consequent DBP impairment. A compensatory rise in CI in response to the drop in BP in T2DM is driven by both elevated absolute HR and lower drops from supine baseline SVI in the diabetes group.

The brain is a metabolically demanding organ that requires around 20% of available oxygenated blood flow at rest (6). Maintaining adequate CBF is crucial for normal brain function as sufficient blood flow is required for the supply of nutrients and oxygen as well as the removal of toxins and other cellular waste products. Studies have previously reported an association between diabetes, lower cerebral perfusion, and deficits in cognitive functioning (27–29). Our results showed that TSI was also lower in T2DM on standing. These results demonstrate that the central hemodynamic response to standing was impaired in diabetes, throughout the experiment in terms of absolute TSI and in the initial recovery period with respect to change from supine baseline TSI, even after controlling for seated blood pressure, arterial stiffness, and use of hypertensives in addition to a number of other possible confounders. CI and TSI are normally positively correlated in healthy individuals; however, our results show that in T2DM, there is an increase in CI from supine baseline and a simultaneous decrease in TSI from supine baseline in response to standing. One possible explanation for this may be that impaired recovery of HR, BP, and hence TPR and possibly impaired cerebral autoregulation are acting in T2DM, and so the consequent increase in CI to compensate for the drop in BP may be insufficient to counteract the drop in TSI. It is noteworthy that the Finometer cannot reliably estimate absolute CI, so it is also possible that absolute CI may be lower in T2DM and it is just the relative change from supine baseline that is increased.

We hypothesized that hypoperfusion maybe 1 potential mechanism linking impaired blood pressure recovery on standing to poorer cognition in diabetes. In the current study, T2DM was associated with lower change from supine baseline TSI in recovery to standing (during the first 60 seconds after standing) as well as lower absolute TSI throughout. We also showed that T2DM and TSI were both associated with cognitive impairment. Our findings suggest that although T2DM and poorer recovery of TSI were both associated with impaired cognition independent of other confounders, we were unable to demonstrate evidence for an interaction effect between the 2. This may be due to sample size limitations or the fact that the association of T2DM with cognitive impairment does not change with TSI. Importantly, the coefficient for T2DM and TSI recovery remained unchanged and significant even after controlling for peripheral hemodynamic responses and so suggests that the effect of cerebral perfusion as measured by TSI recovery is associated with cognitive impairment independent of these peripheral hemodynamic responses.

We observed a gradient in the response to standing from No Diabetes to Pre-Diabetes to T2DM across nearly all hemodynamic responses studied (particularly evident in absolute HR) with T2DM having the most impaired response to orthostasis across all signals. One possible explanation for this is that the impairments and gradients observed between pre-diabetes and diabetes, particularly in the peripheral hemodynamic responses to standing, may be indicative of early signs of autonomic neuropathy, a severe complication of diabetes that results in impairment of autonomic control of the cardiovascular system and whose prevalence has been reported to be as high as 60% in patients with long-standing T2DM (53).

Although more investigation is needed to determine whether the statistical significance of this work translates to clinical significance in older adults with T2DM, these findings and the fact that T2DM has statistically significant different features of peripheral and cerebral autonomic functioning which persist after robust covariate adjustment demonstrates that subtle impairments in peripheral and central hemodynamic responses are present in T2DM and may explain some of the association between T2DM and cerebral perfusion and cognitive impairment. Importantly, the population under study was a younger-old population, with a mean age of 64 years. T2DM is a risk factor for later cognitive decline during the fourth to sixth decades of life, and although our study is cross-sectional in nature, it hints that subtle autonomic dysfunction in T2DM may be associated with cognitive dysfunction in older adults with T2DM.

One of the major strengths of this study is that this is, to our knowledge, the first time that the link between diabetes, peripheral hemodynamic, and cerebral oxygenation measured by TSI using NIRS during orthostasis has been investigated and reported. Additionally, this is also the first time that functional regression analyses have been used to allow for flexible high-resolution modeling of the peripheral and central hemodynamic responses to standing. This allows for the investigation of the independent dynamic association between diabetes and central and peripheral hemodynamic responses after robustly controlling for confounding factors. This allowed us to adjust for known covariates at all time points, which have important known impacts on hemodynamic responses to orthostasis.

Some limitations were also present. The TILDA participants who were able/willing to partake in the active stand test were in general higher educated and a healthier subsample of the larger study. Therefore, the estimated effect size for diabetes and other confounders may have been underestimated due to this sample bias. Another limitation is that NIRS only measures cerebral oxygenation of the left frontal lobe. Therefore, our findings cannot be extended to other areas of the brain or to global cerebral blood flow as it may be possible that impaired cerebral perfusion in diabetes is localized to the frontal lobe due to a redistribution of oxygen supply to protect other essential functional brain areas. This analysis was cross-sectional; therefore, causal effects cannot be inferred from any analyses shown. It should also be noted that the TILDA study lacks racial diversity but was representative of 1 in 156 community-dwelling adults aged 50+ in Ireland at its baseline wave.

In summary, we found that T2DM was associated with impaired peripheral and central hemodynamic responses to orthostasis in older adults in multivariate analysis. Further, T2DM and cerebral oxygenation, measured via TSI recovery during orthostasis, were associated with a greater likelihood of impaired cognitive function in community-dwelling older adults, and TSI recovery attenuated the effect of diabetes in the model for cognitive impairment. Importantly, these findings were demonstrated using detailed functional regression analysis allowing for interpretable visualization of these findings at all time points during active stand. Our findings demonstrate the striking differences in T2DM with respect to peripheral and central hemodynamic responses to standing in a population of (relatively young) older adults and identify the precise time when T2DM differs significantly from no diabetes. We also show that T2DM and TSI are both associated with cognitive impairment and that those with T2DM have both an impaired response to active stand and a lower overall level of absolute TSI in general. This hints that subtle autonomic dysfunction manifested as impaired peripheral and central hemodynamic responses may be a potential link between T2DM and cognitive impairment in community-dwelling older adults.

## Conclusion

This study represents the first comprehensive assessment of peripheral and central hemodynamic responses to orthostasis in T2DM and may offer a potential mechanism linking T2DM and adverse brain health outcomes, such as cognitive impairment and dementia in older adults with T2DM.

## Supplementary Material

glae073_suppl_Supplementary_Material

## Data Availability

The dataset(s) supporting the conclusions of this article are not publicly available due to data protection regulations but are accessible at TILDA on reasonable request. The procedures to gain access to TILDA data are specified at https://tilda.tcd.ie/data/accessing-data/.
