# Supplementary material for: The Impact of Type 2 Diabetes on Peripheral and Cerebral Hemodynamic Responses to Active Stand
Source: J Gerontol A Biol Sci Med Sci. 2024 Mar 4;79(5):glae073. doi: 10.1093/gerona/glae073 (PMC11025558; doi:10.1093/gerona/glae073)
Supplement: glae073_suppl_Supplementary_Material [file glae073_suppl_supplementary_material.docx]

**Appendix 1:** **Active Stand Experiment and Feature Description**

***Measurement of Peripheral and Central Haemodynamic Responses to Orthostasis***

In order to assess haemodynamic responses to an active stand (orthostasis), participants lay supine for ~10 minutes before standing. They then remained standing for 3 minutes before completion of the test. The active stand was supervised by a research nurse who assisted with standing as necessary.

A Finometer MIDI device (Finapres Medical Systems BV, Amsterdam, the Netherlands) was used to simultaneously measure continuous beat-to-beat measurements of Systolic/Diastolic Blood Pressure (SBP/DBP), Heart Rate (HR), Total Peripheral Resistance (TPR), Stroke Volume (SV) and Cardiac Output (CO). A finger cuff was placed on the middle or proximal phalanx of a finger (as per manufacturer instructions) on the left hand. Calibration was performed using Physiocal™, while hydrostatic pressures differences were corrected for using the system's height correction unit. The left arm was placed in a sling at heart level to reduce hand movement during transition to stand.

Simultaneous measurements of cerebral oxygenation were captured using a NIRS device during active standing (Portalite; Artinis Medical Systems, Zetten, the Netherlands). The device optode was affixed to the forehead in approximately the FP1/left frontal lobe position of the ten-twenty electrode system (3 cm lateral and 3.5 cm superior to the nasion) (1). The NIRS device recorded relative changes in oxyhemoglobin O2Hb (μM) and deoxyhemoglobin concentration HHb (μM) to the left frontal lobe at 50Hz throughout the active stand experiment. The Tissue Saturation Index (TSI), derived using Spatially Resolved Spectroscopy (SRS) was calculated as the ratio of oxygenated to total haemoglobin, expressed as a percentage. The stand time was identified using a built-in height sensor in the Finometer device and stand time for the NIRS signals was subsequently aligned to the Finometer data (2).

In all cases, the active stand protocol was conducted in a temperature-controlled room maintained between 21°C and 23°C and a black headband was used to cover the NIRS sensor to minimise the influence of ambient light and device motion. Signals were extracted using MATLAB (R2019b) and down-sampled to 1Hz prior to analysis, providing 210 datapoints for each neuro-cardiovascular signal per participant (1 data point per second). For further information on the active stand see elsewhere (3-5).

**References**

1. Klem GH et al. The ten-twenty electrode system of the International Federation. The International Federation of Clinical Neurophysiology. Electroencephalogr Clin Neurophysiol Suppl. 1999;52:3–6.

2. O’Connor JD et al. Impaired Stabilization of Orthostatic Cerebral Oxygenation Is Associated With Slower Gait Speed: Evidence From The Irish Longitudinal Study on Ageing. J Gerontol A Biol Sci Med Sci. 2022;77(6):1216–21.

3. Soraghan C et al. TILDA Signal Processing Framework (SPF) for the analysis of BP responses to standing in epidemiological and clinical studies. In: IEEE-EMBS International Conference on Biomedical and Health Informatics (BHI). 2014. p. 793–6.

4. Knight S, et al. Associations between Neurocardiovascular Signal Entropy and Physical Frailty. Entropy. 2021;23(4).

5. Finucane C et al. A practical guide to active stand testing and analysis using continuous beat-to-beat non-invasive blood pressure monitoring. Clin Auton Res. 2019 Aug;29(4):427-441.

**Appendix 2 Sample size and exclusion criteria**

Overall, 4,115 participants completed the health assessment and initially agreed to take part in the active stand experiment using the Finometer. Of these, 3 participants later removed their consent for their data to be used; 367 were excluded due to technical issues, 223 were unable to participate in the active stand protocol, 188 had invalid/missing data, 182 did not partake in the blood test and so did not have HbA1c data and 168 did not have physical activity data; leaving 2,984 participants included in the current analyses. Of 3,570 participants who initially agreed to partake in the NIRS testing of the active stand experiment a total of 2,496 had valid data and were included in the current analysis. See eTable 1 for a full list of exclusion criteria for the Finometer and NIRS data.

**eTable 1:** **Exclusion criteria and sample sizes for peripheral and central hemodynamic data**

| Description | Peripheral Hemodynamic Data (Finometer) | Central Hemodynamic Data (NIRS) |
| --- | --- | --- |
| Total number of participants in Wave 3 TILDA who took part in the computer assisted personal interview and health assessment and had data for the active stand experiment | 4115 | 3570 |
| Initially took part but later removed consent to use data | 3 | 3 |
| Exclusion Criteria |  |  |
| Technical equipment/software/processing issues | (367) | (532) |
| Participant Unable/Unwilling to participate | (223) | (126) |
| Signals with Missing/Invalid data | (188) | (200) |
| No HbA1C values/didn’t participate in blood test | (182) | (112) |
| Missing IPAQ data | (168) | (101) |
| Total Sample Included | **2984** | **2496** |

**Appendix 3: Variable Definitions**

**Medication Use**

To account for medications potentially influencing haemodynamic responses to active standing, the following medications were identified using ATC codes: beta-blockers (C07AS, C07AG), calcium channel blockers (C08C, C08D, C08E, C08G), other antihypertensives included: thiazides (C03A), alpha-adrenoceptor antagonists (C02CA, C02LE), angiotensin-converting enzyme inhibitors (C09A, C09B) or angiotensin-II receptor blockers (C90C, C09D).

Lipid modifying medications used to treat high cholesterol included: lipid-modifying agents (excluding omega-3-triglycerides) C10 (excluding C10AX06).

Antidepressants, anti-psychotics and anxiolytics (including benzodiazepine and related drugs) were also identified (N05BA, N05AE, N05CD, N05CF, N06A, N05A) based on known effects on orthostatic blood pressure behaviour (3-5).

### **Other Confounders**

Demographic and clinical variables obtained as part of routine assessment included age (continuous), sex and education (primary, secondary, third), waist-to-height ratio (continuous), self-reported smoking status (never, past smoker, current smoker) and self-reported physical activity using the International Physical Activity Questionnaire (inactive, moderate, vigorous).

Presence of a cardiovascular disease excluding hypertension (binary Yes/No) was obtained by self-reported diagnosis of any of: atrial fibrillation, angina, heart attack, heart failure, heart murmur or arrhythmia. Hypertension was not included in this definition as it is separately identified under medication usage above.

The number of medical comorbidities was calculated from self-reported doctor’s diagnoses of the following: cataracts, glaucoma, age related macular degeneration, lung disease, asthma, arthritis, osteoporosis, cancer, Parkinson’s Disease, stomach ulcer, liver disease, and categorised as 0,1 or 2+ comorbidities.

Pulse wave velocity (m/s) was measured as part of the health assessment using a Vicorder®, a measurement of arterial stiffness with the average of two measurements between carotid and femoral arteries obtained.

Seated systolic blood pressure was calculated as the average from two readings taken one minute apart using an Omron™ digital oscillometric BP monitor.

Disability was measured by creating a binary variable to indicate difficulties with any instrumental activity of daily living (IADL) (using the telephone, managing money, taking medication, shopping, and preparing meals) and difficulties with any activity of daily living (ADL) (walking across the room, dressing, bathing, eating, getting in or out of bed, and using the toilet).

**eTable 2: Characteristics of additional covariates according to diabetes status**

| Physical Activity n (%) |  |  |  | 0.003 |
| --- | --- | --- | --- | --- |
| *Inactive* | 769 (31.6) | 139 (38.7) | 79 (41.8) |  |
| *Moderate* | 966 (39.7) | 116 (32.3) | 66 (34.9) |  |
| *Vigorous* | 697 (28.7) | 104 (29.0) | 44 (23.3) |  |
| Medications |  |  |  |  |
| *Beta Blockers*  *n (%)* | 255 (10.5) | 77 (21.4) | 56 (29.6) | <0.001 |
| *Calcium Channel Blockers n(%)* | 221 (9.1) | 54 (15) | 37 (19.6) | <0.001 |
| *Other Antihypertensives n(%)* | 351 (14.4) | 77 (21.4) | 68 (36) | <0.001 |
| *Lipid Modifying Medication n (%)* | 708 (29.1) | 182 (50.7) | 129 (68.3) | <0.001 |
| *Antidepressant/ anxiolytic or Antipsychotic Medication n (%)* | 221 (9.1) | 26 (7.2) | 25 (13.2) | 0.068 |
| Cardiovascular Conditions (excl. hypertension) |  |  |  | <0.001 |
| *0* | 2054 (84.5) | 271 (75.5) | 135 (71.4) |  |
| *1+* | 378 (15.5) | 88 (24.5) | 54 (28.6) |  |
| Other comorbidities |  |  |  | 0.064 |
| 0 | 1182 (48.6) | 169 (47.1) | 76 (40.2) |  |
| 1 | 788 (32.4) | 108 (30.1) | 65 (34.4) |  |
| 2+ | 462 (19.0) | 82 (22.8) | 48 (25.4) |  |
| Disabilities |  |  |  | 0.064 |
| 0 | 2336 (96.1) | 337 (93.9) | 177 (93.7) |  |
| 1+ | 96 (3.9) | 22 (6.1) | 12 (6.4) |  |

**Note.** Cardiovascular Comorbidities includes presence of any of the following: atrial fibrillation, angina, heart attack, heart failure, heart murmur or arrhythmia. Other comorbidities include a count (0,1,2+) of any of the following: cataracts, glaucoma, age related macular degeneration, lung disease, asthma, arthritis, osteoporosis, cancer, Parkinson’s Disease, stomach ulcer, liver disease.

**Cognitive Impairment**

The Montreal Cognitive Assessment (MoCA) was used as an assessment of global cognitive performance, with a score of 23 or less classified as impaired cognitive performance based on published normative population data (6).

**References**

1. American Diabetes Association T. Classification and diagnosis of diabetes. Diabetes Care. 2017;40 (Supple(January):S11–24.

2. Leahy S et al. Prevalence and correlates of diagnosed and undiagnosed type 2 diabetes mellitus and pre-diabetes in older adults: Findings from the Irish Longitudinal Study on Ageing (TILDA). Diabetes Res Clin Pr. 2015;110(3):241–9.

3. Dyer A et al, Study Group N. Antidepressant use and orthostatic hypotension in older adults living with mild-to-moderate Alzheimer disease. Int J Geriatr Psychiatry. 2020;35(11):1367–75.

4. Briggs R et al. The association between antidepressant use and orthostatic hypotension in older people: a matched cohort study. J Am Soc Hypertens. 2018;12(8):597–604.

5. Rivasi G et al. Effects of benzodiazepines on orthostatic blood pressure in older people. Eur J Intern Med. 2020;72:73–8.

**6.** Thomann AE et al. Enhanced diagnostic accuracy for neurocognitive disorders: A revised cut-off approach for the Montreal Cognitive Assessment. Alzheimer’s Res Ther. 2020;12(1):1–10.

**Appendix 4: Function on Scalar Regression to model Haemodynamic Responses to Standing**

To model the response to standing of HR, SBP, DBP, TPR, SV, CO and TSI independently, function-on-scalar regressions were implemented using the haemodynamic curves as the response variable (56).

More specifically, the participants’ response curves $\boldsymbol{y}\left( t \right)=\left( y_{1}\left( t \right),\ldots,y_{N}\left( t \right) \right)^{T}$, measured at time points $t=1, \ldots,1051$, were first smoothed by exploiting a set of B-splines basis functions $\boldsymbol{\theta}\left( t \right)=\left( \theta_{1}\left( t \right),\ldots,\theta_{k}\left( t \right) \right)^{T}$. We then assume the following model:

$$\boldsymbol{y}\left( t \right)=\boldsymbol{Z}\boldsymbol{\beta}\left( t \right)+\boldsymbol{\epsilon}\left( t \right)$$

$Z$ is an $N \times q$design matrix and the regression coefficient functions $\beta\left( t \right)=\left[ \beta_{1}\left( t \right)\ldots\beta_{q}\left( t \right) \right]^{T}$ are assumed to lie in the span of $\boldsymbol{\theta}(t)$. Finally, $\boldsymbol{\varepsilon}\left( t \right)=\left( \varepsilon_{1}\left( t \right),\ldots,\varepsilon_{N}\left( t \right) \right)^{T}$ is a vector of random error functions with independent components and such that the expected value of $\varepsilon_{i}\left( t \right)$ is zero for any $i$ and $t$. Here we have $N=2989$ participants for peripheral haemodynamic responses and $N=1935$ for central oxygenation responses (See Appendix 1 for a breakdown of exclusion criteria). The number of basis coefficients was set as $K$=40. The number of bases $K$ and the smoothness parameter $\lambda$ were selected through generalised cross validation. A sensitivity analysis using a grid search of these parameters noted the generalised cross validation error was low and very similar over a wide range of values for K and $\lambda$. Hence these values were tuned for computational parsimony. Appendix 2 shows the results of a grid search for $\lambda$ and the stability of the results for heart rate when varying the values of K from 10 to 150 which informed our choice. Bases were evenly spaced. However, sensitivity analyses showed that changing the position of the bases did not substantially change the results and did not affect any conclusions drawn.

**Appendix 4.1: Choice of smoothness parameter and number of bases**

Generalised cross validation was used to inform the choice of values for the smoothing parameter $\lambda$ and number of bases K used to smooth the neuro-cardiovascular curves modelled in this study using B-spline basis expansion. The level of smoothing and number of bases were chosen using generalized cross-validation criterion $GCV\left( \lambda\right)$:

$$GCV\left( \lambda\right)=\frac{1}{n}\sum_{i}^{n} \left( y_{i}-\hat{y}_{i}^{\lambda} \right)^{2}w_{i}\left( 1-tr\left( S_{\lambda} \right)n^{-1} \right)^{-2}$$

Where n is the number of timepoints, $y_{i}$ is the value of the curve at point $i$, $w_{i}$ is the weight at point $t\_i$ and $S_{\lambda}$ is the smoothing matrix defined by ${f_{\lambda}=S}_{\lambda}y$. Figure 1 below shows the GCV for a range of values of $\lambda$ and K. Here it can be seen that the GCV does not change substantially with respect to $\lambda$ when K<80 hence a large value of $\lambda=500 was selected$ for computational simplicity.


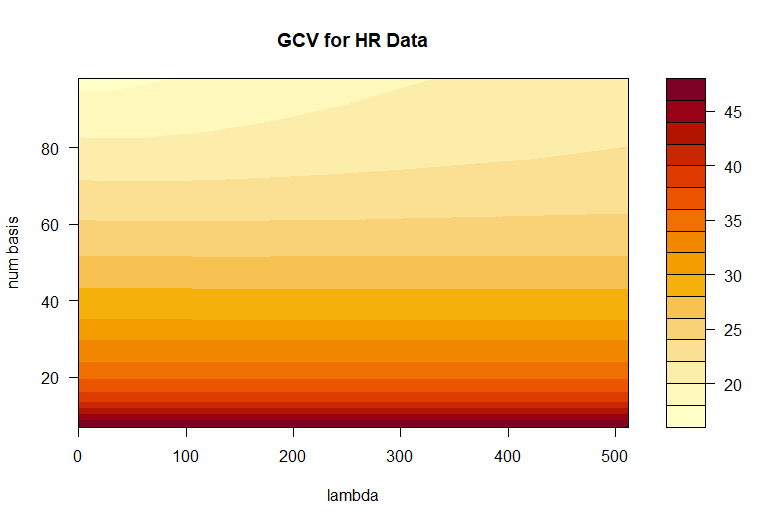


eFigure 1: Generalised Cross Validation error for values of $\lambda$=[0,500] and K=[10,20,40,50,60,80,100,150]

Figure S2 shows boxplots of the coefficient values of the explanatory variables of heart rate when ranging the number of bases K from 10 to 150. Here it can be seen that the results are very stable across the range of values of K and that in particular results from K=40 to 150 are incredibly stable. Hence for parsimony K=40 and was selected $\lambda=500$ were selected as the final results reported in the main analysis. Similar results were observed for all other haemodynamic curves studied and choice of parameters did not affect the conclusions of the study.

eFigure 2: Boxplot of coefficient values for model of heart rate when ranging K from 10 to 150 . This shows that the coefficients in all cases were stable regardless of the number of basis chosen.

**Appendix 5: Functional Regression Coefficients**

eFigure 3: Functional Coefficients of model of heart rate (change from baseline). Solid lines denote the mean coefficient estimate and shaded areas the pointwise 95% confidence intervals. In each case the x-axis indicates the timeline of the active stand experiment from 30 seconds before stand (denoted -30) to 180 seconds after stand. The standpoint is denoted as time 0 in all cases. Time points where the confidence intervals do not cross zero on the y-axis are statistically significant.

NOTE : For illustrative purposes and to ease interpretation, we will further explain the intercept term for heart rate (Figure 1 C). This is the average change from baseline heart rate response which would be expected if all other coefficients are zero. Here it can be seen that the change from baseline heart rate is 0 bpm from 30 seconds before stand to the standpoint at time 0 (when all other coefficients are set to zero). Upon standing, it increases to a peak of 29 bpm 12 seconds after stand and hits a post stand minimum at 23 seconds after stand.

In a similar vein, it can be seen, older age is associated with lower change from baseline heart rate in the recovery period 90-180 seconds after standing. Females have significantly lower heart rate compared to males. Each unit increase in pulse wave velocity was significantly associated with on average 0.11 bmp higher heart rate throughout the experiment, while use of betablockers were associated with significantly lower heart rate throughout. Higher seated systolic blood pressure was negatively associated with heart rate post stand but not at rest.

eFigure 4: *Functional Coefficients of model of delta systolic blood pressure. Solid lines denote the mean coefficient estimate and shaded areas the pointwise 95% confidence intervals.*

eFigure 5: *Functional Coefficients of model of delta diastolic blood pressure. Solid lines denote the mean coefficient estimate and shaded areas the pointwise 95% confidence intervals.*

eFigure 6: *Functional Coefficients of model of delta total peripheral resistance. Solid lines denote the mean coefficient estimate and shaded areas the pointwise 95% confidence intervals.*

eFigure 7 *Functional Coefficients of model of delta stroke volume. Solid lines denote the mean coefficient estimate and shaded areas the pointwise 95% confidence intervals.*

eFigure 8: *Functional Coefficients of model of delta cardiac output. Solid lines denote the mean coefficient estimate and shaded areas the pointwise 95% confidence intervals.*

eFigure 9: *Functional Coefficients of model of delta TSI. Solid lines denote the mean coefficient estimate and shaded areas the pointwise 95% confidence intervals.*

eFigure 10: *Functional Coefficients of model of absolute HR. Solid lines denote the mean coefficient estimate and shaded areas the pointwise 95% confidence intervals*

eFigure 11: *Functional Coefficients of model of absolute TSI. Solid lines denote the mean coefficient estimate and shaded areas the pointwise 95% confidence intervals*


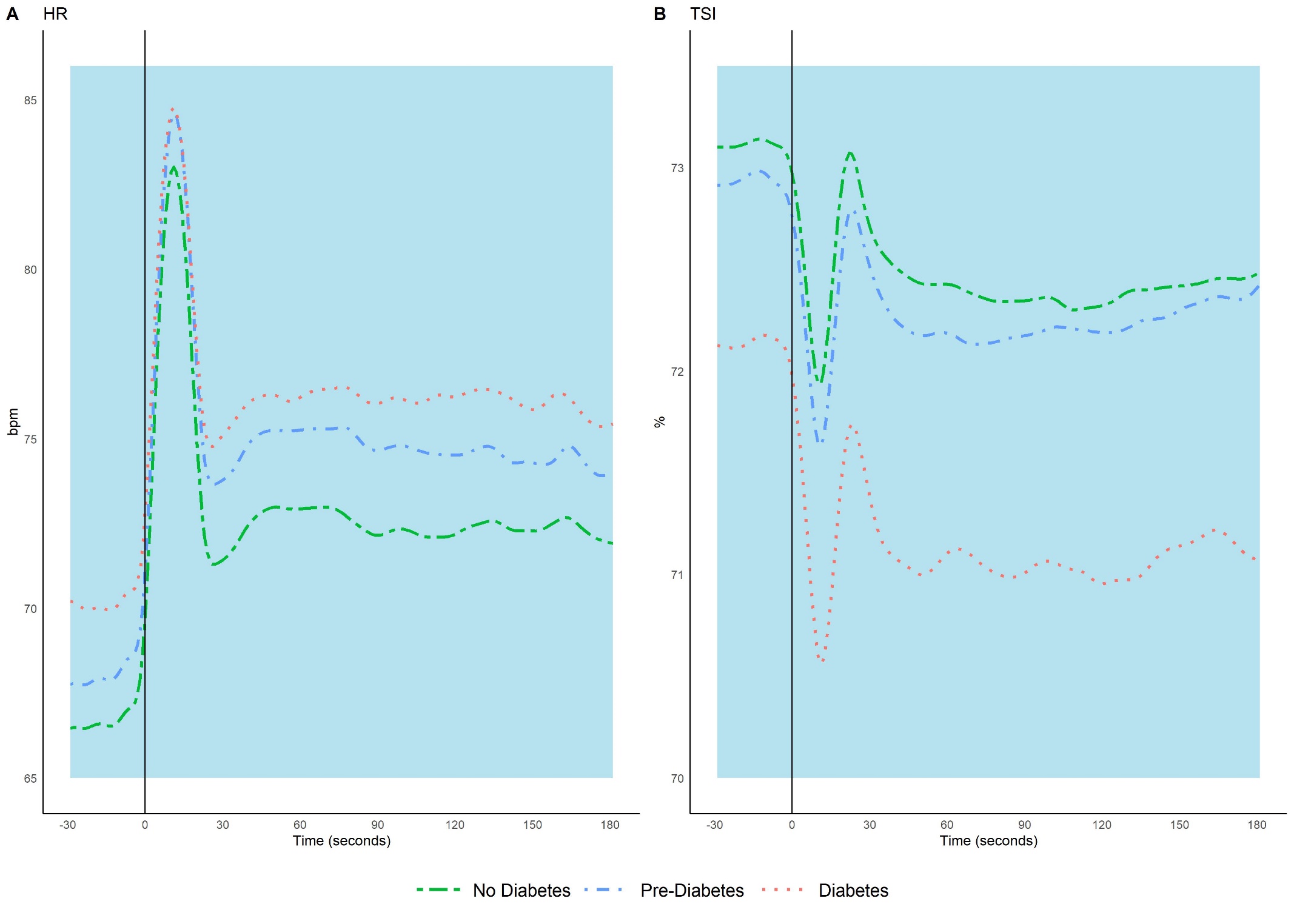


eFigure 12: Marginal Effects of diabetes group on absolute HR and absolute TSI. Note: Change from baseline was taken for other signals as the finometer cannot reliably capture absolute values of SBP, DBP, SVI, CI or TPR.

**eTable 2. Association Between Diabetes Status, Peripheral and Haemodynamic Responses to Active Stand and Impaired Cognitive Performance**

| A |  |  |  |
| --- | --- | --- | --- |
| Coefficient | **Odds Ratio** | **95% CI** | **p-value** |
| Pre-*Diabetes* | 1.07 | (0.77,1.46) | 0.688 |
| *Diabetes* | 1.62 | (1.07,2.41) | 0.019 |
| *Mean TSI Recovery* | 0.90 | (0.81,0.99) | 0.047 |
| *B* |  |  |  |
| Coefficient | **Odds Ratio** | **95% CI** | **p-value** |
| Pre-*Diabetes* | 1.11 | (0.80,1.52) | 0.540 |
| *Diabetes* | 1.61 | (1.03,2.47) | 0.034 |
| *Mean TSI Recovery* | 0.89 | (0.80,0.99) | 0.037 |
| *Mean HR Recovery* | 0.98 | (0.85,1.12) | 0.772 |
| *Mean SBP Recovery* | 1.01 | (0.80,1.28) | 0.949 |
| *Mean DBP Recovery* | 1.02 | (0.74,1.41) | 0.917 |
| *Mean CI Recovery* | 1.06 | (0.91,1.24) | 0.442 |
| *Mean TPR Recovery* | 0.95 | (0.77,1.14) | 0.579 |

**Note**. Odds ratios, 95% confidence intervals and p-values from logistic regression of cognitive impairment. For all signals Mean Recovery refers to the coefficient for the mean signal during the recovery period (90-180 seconds post stand). Results are after adjustment for age, sex, education, comorbidities, use of anti-depressants/anti-psychotic and/or anxiolytic medication, seated hypertension and/or use of anti-hypertensive medication, physical activity, smoking status, presence of disabilities and waist to height ratio. Table 3 B: Results from a model including all of the covariates in the model represented in Table A and also additionally adjusting for mean peripheral haemodynamic signals during recovery phase.
